# Minnesota Medical Directors: Challenges and Opportunities

**DOI:** 10.1093/geroni/igaf122.3031

**Published:** 2025-12-31

**Authors:** Rajean Moone, Teresa McCarthy, Megan Hakanson

**Affiliations:** University of Minnesota, Minneapolis, Minnesota, United States; University of Minnesota, Minneapolis, Minnesota, United States; University of Minnesota, Minneapolis, Minnesota, United States

## Abstract

Nursing homes must appoint a physician as a Medical Director, yet little is known about their education, challenges, and needs. We conducted an online survey to assess the credentials, responsibilities, and difficulties faced by Minnesota Medical Directors. Since no comprehensive list existed, we called all Minnesota nursing homes to identify their Medical Directors. At 147 facilities (43%), staff were unaware of who held this role. A total of 26 Medical Directors, representing 42% of state nursing homes, responded. Most were male, white, and had served an average of 14 years, spending about six hours on-site monthly. Only 19% completed a geriatrics fellowship, and 46% held a medical director certification. Employment status varied: 69% with a nursing home, 42% through a medical group, and 4% via a stipend. All participated in their facility’s quality committee. Qualitative analysis revealed 11 key challenges, including staffing shortages, communication issues, excessive regulations, clinical complexity, leadership struggles, and time constraints. Four main needs emerged: additional education, peer support, regulatory consistency, and more time for patient care. As Medical Directors’ roles evolve, understanding their needs and challenges is crucial to enhancing support and improving nursing home care.

